# Biosynthesis of Polyhydroxyalkanoate from Steamed Soybean Wastewater by a Recombinant Strain of *Pseudomonas* sp. 61-3

**DOI:** 10.3390/bioengineering4030068

**Published:** 2017-08-08

**Authors:** Ayaka Hokamura, Yuko Yunoue, Saki Goto, Hiromi Matsusaki

**Affiliations:** Department of Food and Health Sciences, Faculty of Environmental and Symbiotic Sciences, Prefectural University of Kumamoto, Kumamoto 862-8502, Japan; a_hokamura@pu-kumamoto.ac.jp (A.H.); aocharirider@yahoo.co.jp (Y.Y.); g1675001@pu-kumamoto.ac.jp (S.G.)

**Keywords:** polyhydroxyalkanoate, PHA, copolymer, soybean wastewater

## Abstract

*Pseudomonas* sp. 61-3 accumulates a blend of poly(3-hydroxybutyrate) [P(3HB)] homopolymer and a random copolymer, poly(3-hydroxybutyrate-*co*-3-hydroxyalkanoate) [P(3HB-*co*-3HA)], consisting of 3HA units of 4–12 carbon atoms. *Pseudomonas* sp. 61-3 possesses two types of PHA synthases, PHB synthase (PhbC) and PHA synthases (PhaC1 and PhaC2), encoded by the *phb* and *pha* loci, respectively. The P(94 mol% 3HB-*co*-6 mol% 3HA) copolymer synthesized by the recombinant strain of *Pseudomonas* sp. 61-3 (*phbC*::*tet*) harboring additional copies of *phaC1* gene is known to have desirable physical properties and to be a flexible material with moderate toughness, similar to low-density polyethylene. In this study, we focused on the production of the P(3HB-*co*-3HA) copolymer using steamed soybean wastewater, a by-product in brewing *miso*, which is a traditional Japanese seasoning. The steamed soybean wastewater was spray-dried to produce a powder (SWP) and used as the sole nitrogen source for the synthesis of P(3HB-*co*-3HA) by the *Pseudomonas* sp. 61-3 recombinant strain. Hydrolyzed SWP (HSWP) was also used as a carbon and nitrogen source. P(3HB-*co*-3HA)s with relatively high 3HB fractions could be synthesized by a recombinant strain of *Pseudomonas* sp. 61-3 (*phbC*::*tet*) harboring additional copies of the *phaC1* gene in the presence of 2% glucose and 10–20 g/L SWP as the sole nitrogen source, producing a PHA concentration of 1.0–1.4 g/L. When HSWP was added to a nitrogen- and carbon-free medium, the recombinant strain could synthesize PHA without glucose as a carbon source. The recombinant strain accumulated 32 wt% P(3HB-*co*-3HA) containing 80 mol% 3HB and 20 mol% medium-chain-length 3HA with a PHA concentration of 1.0 g/L when 50 g/L of HSWP was used. The PHA production yield was estimated as 20 mg-PHA/g-HSWP, which equates to approximately 1.0 g-PHA per liter of soybean wastewater.

## 1. Introduction

Polyhydroxyalkanoates (PHAs) are accumulated in many bacteria as intracellular carbon and energy storage materials under nutrient-limited conditions in the presence of excess carbon [1,2,3]. PHAs are regarded as important environmentally compatible materials because of their potential use as biodegradable plastics with properties similar to petroleum-based plastics. PHAs can be divided into three groups based on their monomer structure. Short-chain-length PHAs (scl-PHAs) consisting of monomers with 3 to 5 carbon atoms, medium-chain-length PHAs (mcl-PHAs) consisting of monomers with 6 to 14 carbon atoms and scl-mcl-PHA copolymers consisting of both scl and mcl monomer units [4]. Poly(3-hydroxybutyrate) [P(3HB)], the principal member of the scl-PHAs, is both stiff and brittle. In contrast, mcl-PHA, consisting of mcl-3-hydroxyalkanoate (mcl-3HA) units with 6 to 14 carbon atoms, is generally amorphous because of its low crystallinity. PHA copolymers consisting of scl and mcl monomers synthesized by recombinant bacteria show various properties ranging from stiff to flexible depending on the monomer composition. Therefore, the composition ratio of scl and mcl monomers is crucial in PHA properties. In particular, the P(94% 3HB-*co*-6% 3HA) copolymer consisting of 3HA units of 6 to 12 carbon atoms possesses properties similar to low-density polyethylene (LDPE) [5]. However, the commercial development of PHAs has been limited because of their high production costs. Therefore, it is desirable that PHAs are economically produced from inexpensive carbon sources such as waste substrates.

In this regard, several studies have reported PHA production using waste substrates as carbon sources. P(3HB) homopolymer has been reported to be synthesized by *Cupriavidus necator* H16 (formerly *Ralstonia eutropha* H16) from plant oils as the sole carbon sources [6]. Furthermore, a random copolymer, P(3HB-*co*-3-hydroxyhexanoate), with a high PHA content has been synthesized from plant oils using a recombinant strain of *C. necator* PHB¯4 (a PHA-negative mutant) harboring the PHA synthase gene from *Aeromonas cavie* [6]. Wong and Lee reported that P(3HB) could be synthesized from whey using the *Escherichia coli* strain GCSC 6576 harboring the PHA-biosynthetic operon from *C*. *necator* and the *ftsZ* gene from *E*. *coli* [7]. In a recent study, P(3HB) homopolymer and a P[3HB-co-3-hydroxyvalerate (3HV)] copolymer have been reported to be produced from waste such as oil extracted from spent coffee grounds [8] and waste from the olive oil industry [9]. *Cupriavidus* sp. KKU38 has been reported to synthesize P(3HB) from cassava starch hydrolysate [10]. A number of reviews on the topic have been published [11,12,13]. While there are numerous reports of the production of scl-PHA or mcl-PHA from waste, there are very few reports on the production of scl-mcl PHA from waste, although Wang et al. have reported scl-mcl PHA production from glycerol, a by-product of the biodiesel industry, using engineered *Escherichia coli* [13]. The production of scl-mcl PHAs consisting of 3HB and mcl-3HA from biomass sources is desirable for the dissemination of PHA as biodegradable plastics because the copolymer is expected to have various properties, ranging from stiff to flexible, depending on the monomer composition as described above.

*Pseudomonas* sp. 61-3 synthesizes two kinds of PHAs, a P(3HB) homopolymer and a random copolymer, P(3HB-*co*-3HA), consisting of 3-hydroxyalkanoate (3HA) units of 4–12 carbon atoms [14,15,16]. *Pseudomonas* sp. 61-3 possesses two types of PHA synthases, PHB synthase (PhbC) and PHA synthases (PhaC1 and PhaC2), encoded at the *phb* and *pha* loci, respectively [16]. PhbC shows substrate specificities for short-chain-length 3HA units, whereas PhaC1 and PhaC2 are able to incorporate a wide range of 3HA units of 4–12 carbon atoms into PHA. It has also been reported previously that PhaC1 is the major PHA providing enzyme in *Pseudomonas* sp. 61-3 [17].

Soybeans are used as raw materials in numerous Japanese foods such as *miso* (fermented soybean pastes), *shoyu* (soy sauce), *natto* (fermented soybean) and *tofu* (soybean protein curd), all of which produce wastewater during the manufacturing process. *Miso* is a traditional Japanese seasoning and many Japanese eat *miso* soup every day. However, the steamed soybean wastewater in produced in *miso* processing is a problem. The wastewater must be treated by a wastewater treatment facility as an activated sludge since the soybean wastewater still contains a large amount of organic compounds, resulting in an enormous cost. Following the production of one ton of *miso*, *shoyu,* or *tofu*, 740, 50, or 18 liters of wastewater is generated, respectively [18]. Their chemical oxygen demand is 32,000, 29,000 and 15,000 ppm, respectively, although they are more than 95% water. In Japan, over 100 million liters of wastewater is generated annually from soybean processed foods such as *miso*, *shoyu*, *natto* and *tofu*. Therefore, the utilization of this soybean wastewater is desirable. For example, there have been several reports describing the recovery of oligosaccharides from steamed soybean wastewater in *tofu* processing [19], the recovery of isoflavone aglycones from soy whey wastewater [20], and the use of the soybean-derived waste as biomass [21,22,23,24]. In this study, PHA production using steamed soybean wastewater as a nitrogen and/or carbon source was performed using a recombinant strain of *Pseudomonas* sp. 61-3. This is the first report describing scl-mcl-PHA production from steamed soybean wastewater.

## 2. Materials and Methods

### 2.1. Preparation and Hydrolysis of Steamed Soybean Wastewater and Starch

Steamed soybean wastewater was collected from barley *miso* (made from barley and soybean) brewery factory in Kumamoto prefecture, Japan and was spray dried to powder. The nutrient composition of the soybean wastewater powder (SWP) was analyzed by Japan Food Research Laboratories, one of the world’s largest and most diversified testing services providers (Table 1). Since SWP contains a sufficient amount of protein, it was first used as a nitrogen source (1, 5, 10, 20 and 50 g/L) for PHA production without further treatment. According to the report by Kimura et al., the constituent sugars of the polysaccharides contained in soybean were arabinose (21.6 wt%), galactose (48.5 wt%), uronic acid (15.0 wt%), and xylose (or rhamnose) (14.9 wt%) [18]. Therefore, polysaccharides that can be used as carbon sources were also considered to be contained in the SWP. In order to investigate the use of a carbon source, an SWP hydrolysate was also prepared using the following two methods. One method involved hydrolysis using 0.6 N H_2_SO_4_ at 80 °C for 5 h. The other hydrolysis method used 5 N H_2_SO_4_ at 90 °C for 1 h. SWP or cornstarch (2.5 g, Kanto Chemical Co., Inc., Japan), as a carbon source control, was treated with 5 mL H_2_SO_4_ (0.6 N or 5 N) for 5 h or 1 h. After hydrolysis, the pH was adjusted to 7.0 using NaOH, and the hydrolysates were subsequently filter-sterilized or autoclaved. The hydrolyzed soybean wastewater powder (HSWP) was used for PHA production as the carbon and the nitrogen source at the concentrations of 10, 20, 30, 40, 50, 75, and 100 g/L. The cornstarch hydrolysate was used as the control of a carbon source.

### 2.2. Bacterial Strain, Plasmid, and Culture Conditions

*Pseudomonas* sp. 61-3 (*phbC*::*tet*), which is a *phbC*-negative mutant [16], and the recombinant strain were grown at 28 °C in a nutrient-broth (NB) medium consisting of 1% meat extract (Kyokuto Pharmaceutical Industrial Co., Ltd., Tokyo, Japan), 1% Bactopeptone (Difco Laboratories, Division of Becton Dickinson Company, Sparks, MD, USA) and 0.5% NaCl (pH 7.0). *Pseudomonas* sp. 61-3 (*phbC*::*tet*) harboring the pJKSc54-*phab*, carrying *phaC1* under the control of the *pha* promoter from *Pseudomonas* sp. 61-3 and *phbAB* under the control of the *phb* promoter from *C. necator*, was used to synthesize P(3HB-*co*-3HA) copolymer as described previously [5]. When needed, kanamycin (50 mg/L) and tetracycline (12.5 mg/L) were added to the medium for plasmid maintenance of this *Pseudomonas* sp. 61-3 recombinant strain.

### 2.3. Production and Analysis of PHA

*Pseudomonas* sp. 61-3 (*phbC*::*tet*) harboring pJKSc54-*phab* was grown on NB medium and transferred to 500 mL shaking flasks containing 100 mL of a nitrogen-free MS medium containing 0.9 g Na_2_HPO_4_·12H_2_O, 0.15 g of KH_2_PO_4_, 0.02 g of MgSO_4_·7H_2_O and 0.1 mL of trace element solution [15]. The culture with an initial absorbance at 600 nm of 0.05 was cultivated on a reciprocal shaker (130 strokes/min) at 28 °C for 48 h or 72 h. SWP as the sole nitrogen source was added to the medium and autoclaved. Filter-sterilized glucose (2 wt%) was aseptically added to the autoclaved medium as the sole carbon source. NH_4_Cl (0.05%), as a control nitrogen source, was also added to the medium instead of SWP. HSWP and hydrolyzed cornstarch were filtered (ADVANTEC filter paper No.1, Toyo Roshi Kaisha, Ltd., Tokyo, Japan) to remove the residues and the filtrates were autoclaved, followed by they were aseptically added to the medium as both the nitrogen and the carbon sources, or the carbon source, respectively. Determination of cellular PHA composition by gas chromatography (GC) was performed as reported previously [13]. Approximately 30 mg of dry cells were subjected to methanolysis and the converted methyl esters were subjected to gas chromatography analysis on a Shimadzu GC-17A system equipped with an Inert Cap 1 capillary column (30 m × 0.25 mm, GL Sciences Inc., Tokyo, Japan) and a flame-ionization detector. Methyl caprylate was used as the internal standard. All cultivations were performed in triplicate.

## 3. Results

### 3.1. Nutrient Composition of SWP

SWP was obtained after spray drying of steamed soybean wastewater obtained from the processing of *miso*. The nutrient composition of SWP is shown in Table 1. Nutrients such as crude protein, crude fat, starch and non-fibrous carbohydrate present in the SWP could therefore be utilized for PHA production as nitrogen/carbon sources.

### 3.2. Utilization of SWP for PHA Production

*Pseudomonas* sp. 61-3 (*phbC*::*tet*) harboring the pJKSc54-*phab* plasmid was cultivated at 28 °C for either 48 h or 72 h in a nitrogen-free MS medium supplemented with 2% glucose and various concentrations (1, 5, 10, 20 and 50 g/L) of SWP as the nitrogen source. P(3HB-*co*-3HA) copolymers were synthesized by the recombinant strain using the SWP as the sole nitrogen source (Table 2). Compared with PHA production using 0.5 g/L of NH_4_Cl as the sole nitrogen source, the PHA content (62 wt%) and the monomer composition of P(3HB-*co*-3HA) synthesized from 1 g/L of SWP were almost same but the dry cell weight (0.4–0.6 g/L) was much lower than the control (NH_4_Cl). Because SWP was considered not to contain sufficient nitrogen source for cell growth, the concentration of SWP added to the medium was then increased. Increasing the concentration of SWP, caused an increase in the dry cell weight, whereas both the PHA content and the 3HB fraction in P(3HB-*co*-3HA) decreased. When the concentration of SWP was increased to 20 g/L or more, the SWP could not be completely dissolved in the medium since a precipitate was observed in the cell pellet after centrifugation of the culture broth. Overall, it was concluded that 10–20 g/L of SWP was suitable for PHA production, and that a PHA concentration of 1.0 to 1.4 g/L was produced. Thus, SWP could be used as the sole nitrogen source instead of NH_4_Cl for P(3HB-*co*-3HA) production by this recombinant strain despite the fact that the PHA concentrations were lower than in the control experiment where 0.5 g/L of NH_4_Cl was used as the sole nitrogen source.

We next investigated whether SWP could be utilized as the carbon source as well as the nitrogen source for the production of P(3HB-*co*-3HA). To achieve this, hydrolysis of SWP was carried out by two methods. First, hydrolysis of SWP was attempted at 80 °C for 5 h with 0.6 N H_2_SO_4_ and the hydrolysate (HSWP) was subsequently used for PHA production as both the carbon/nitrogen source. As a result, the recombinant strain accumulated P(3HB-*co*-3HA) using this HSWP (Table 3). The P(3HB-*co*-3HA) produced by the recombinant strain reached levels of 1.3 g/L using 75 g/L of HSWP. Similar to what was observed for SWP, the 3HB fraction of P(3HB-*co*-3HA) decreased with increasing concentration of HSWP. The composition ratio of scl- and mcl-monomers has a considerable influence on PHA qualities, in particular P(3HB-*co*-3HA) with too low a 3HB fraction is an amorphous polymer [15]. At a concentration of 20 to 75 g/L of HSWP, the recombinant strain accumulated 32–35 wt% PHA, which is a relatively high PHA content.

Subsequently, SWP was hydrolyzed at 90 °C for 1 h with 5 N H_2_SO_4_ to hydrolyze it completely and the hydrolysate was used as the nitrogen and carbon source for PHA production in a similar manner as described above. The recombinant strain accumulated 26 wt% P(3HB-*co*-3HA) and produced 0.2–0.3 g/L of PHA from 10–20 g/L of HSWP (Table 4). However, PHA was barely produced at 40–50 g/L of HSWP, and the recombinant strain no longer grew at 75 g/L or more HSWP; this is likely due to the high concentration of salts formed by neutralization after hydrolysis. In conclusion, it was found that HSWP could be utilized as both the nitrogen and carbon source. In addition, the data suggested that hydrolysis of SWP using 0.6 N H_2_SO_4_ was better than using 5 N H_2_SO_4_ for the cell growth and PHA production.

We attempted to produce PHA from hydrolyzed cornstarch as a carbon source to compare with SWP, using 0.05% NH_4_Cl as the nitrogen source. First, hydrolysis of cornstarch (10 g/L) was attempted at 80 °C for 5 h with 0.6 N H_2_SO_4_. However, no cell growth was observed, which was attributed to the lack of time for hydrolysis or the lack of an amount of cornstarch added to the medium (Table 5). Therefore, hydrolysis of cornstarch (100 g/L) was subsequently performed for 8 h. As a result, the recombinant strain accumulated 71 wt% P(3HB-*co*-3HA) containing 86 mol% 3HB and 12 mol% 3HA (C_6_-C_12_) units from 100 g/L of hydrolyzed cornstarch (Table 5). Additionally, the recombinant strain grew well and the PHA produced reached levels of 2.9 g/L, which was the highest production level among the P(3HB-*co*-3HA)s reported so far. The recombinant strain also grew well and produced 54–63 wt% P(3HB-*co*-3HA) with high 3HB fraction (83–84 mol%) from cornstarch (14 and 20 g/L) hydrolyzed using 5 N H_2_SO_4_ (Table 5), whereas the cell growth and accumulated PHA content were slightly lower than the control using 2% glucose (Table 2).

Finally, PHA production using a mixture of SWP/HSWP and hydrolyzed cornstarch was attempted (Table 6 and Table 7). The recombinant strain accumulated 64 wt% P(3HB-*co*-3HA) containing 82 mol% 3HB (0.3 g/L of PHA concentration) when 1 g/L of SWP and 13 g/L of hydrolyzed cornstarch were added to the medium as the nitrogen and the carbon sources, respectively (Table 6). From 10 g/L of SWP and 13 g/L of hydrolyzed cornstarch, the dry cell weight increased to 1.2 g/L and 0.5 g/L of PHA was obtained. However, the molar fraction of 3HB unit in the copolymer was relatively low (65 mol% 3HB). In the case where 26 g/L of hydrolyzed cornstarch was added, the dry cell weight and the PHA content decreased, resulting in a low PHA concentration (less than 0.2 g/L). When both HSWP (SWP hydrolyzed using 0.6 N H_2_SO_4_) and hydrolyzed cornstarch (hydrolyzed using 5 N H_2_SO_4_) were added to the medium, cell growth was inhibited, except when 50 g/L of HSWP only was used as the sole nitrogen and carbon source (Table 7).

## 4. Discussion

In order to effectively produce PHAs, they should be produced from inexpensive carbon sources such as waste substrates. For this reason, we elected to focus on the use of steamed soybean wastewater generated as a by-product of the soybean processing industry. In this study, P(3HB-*co*-3HA) was synthesized from SWP, HSWP and/or hydrolyzed cornstarch as the nitrogen and/or the carbon sources using *Pseudomonas* sp. 61-3 (*phbC*::*tet*) harboring the *phaC1* gene from *Pseudomonas* sp. 61-3 and *phbAB* genes from *C. *necator**. *Pseudomonas* sp. 61-3 (*phbC*::*tet*) harboring pJKSc54-*phab* accumulated the P(3HB-*co*-3HA) copolymer from SWP and glucose as the sole nitrogen and carbon sources, respectively, but the 3HB fraction in the copolymer decreased at the amount of added SWP increased. This would be responsible for the expression level of PHA synthase gene (*phaC1*) of *Pseudomonas* sp. 61-3. The additional copies of the *phaC1* gene have been previously reported to result in an increase in the 3HB fraction in the copolymer synthesized when glucose was used as the sole carbon source [5]. This is due to the low substrate specificity of PhaC1 for (*R*)-3HB-CoA. PhaC1 synthase has been reported to have the highest activity toward (*R*)-3-hydroxydecanoate (3HD)-CoA among the C_4_-C_12_ substrates [25]. Therefore, a decrease in the expression level of the *phaC1* gene leads to an increase in the 3HA fraction, especially with the 3HD unit being the main component, in the copolymer synthesized through *de novo* fatty acid synthesis pathway when unrelated carbon sources such as glucose were used. To obtain a higher 3HB composition in the copolymer, additional copies of *phaC1* are required, together with the *phbAB* genes, since PHA synthase activity has been reported to affect monomer composition in the copolymer as well as monomer supply by PhbA and PhbB when sugars were used as the sole carbon source [5]. In contrast, when fatty acids were used as carbon source, the 3HO (3-hydroxyoctanoate) fraction increased in the copolymer synthesized by a recombinant *Pseudomonas* sp. 61-3 (*phbC*::*tet*) strain carrying an additional *phaC1* gene compared with the strain containing only the vector [5]. The *phaC1* gene would be expected to be expressed under nitrogen-limited conditions. A sequence resembling the consensus sequence of the *Escherichia coli* σ^54^-dependent promoter involved in expression under nitrogen-limited conditions has been found upstream of the *phaC1* gene [16] and P(3HB-*co*-3HA) was synthesized by *Pseudomonas* sp. 61-3 only under nitrogen-limited conditions [14]. When the initial molar ratio of nitrogen and carbon sources (C/N) was low, the copolymer content was also low [5]. The content (45 wt%) and the concentration (1.13 g/L) of PHA accumulated by this recombinant strain with a C/N molar ratio of 71 were the highest obtained, as reported in a previous study [5]. Thus, limitation of nitrogen source is necessary for the biosynthesis of P(3HB-*co*-3HA) by recombinant strains of *Pseudomonas* sp. 61-3, although nitrogen is essential for bacterial growth. In order to synthesize P(3HB-*co*-3HA) with a high 3HB fraction, similarly, a high expression level of *phaC1* is required. In fact, introduction of only the *phaC1* gene into *Pseudomonas* sp. 61-3 (*phbC*::*tet*) increased the 3HB fraction in P(3HB-*co*-3HA) from 27 mol% to 55 mol% [5]. As the amount of SWP added to the medium increased, that is, as the C/N molar ratio decreased, the 3HB fraction in the copolymer decreased (Table 2). This indicates that SWP can be utilized as a nitrogen source for PHA production by the recombinant *Pseudomonas* sp. 61-3 strain.

In this study, we also attempted to use HSWP for PHA production. Acid hydrolysis of SWP was attempted using two methods; either 0.6 N or 5 N H_2_SO_4_. As a result, 20-75 g/L of HSWP prepared using 0.6 N H_2_SO_4_, resulted in the synthesis of 32–35 wt% P(3HB-*co*-3HA) at the levels of 0.4–1.3 g/L of PHA (Table 3). However, the 3HB fraction in the copolymer decreased with increasing HSWP concentration, presumably due to the low C/N molar ratio as described previously [5]. With regard to PHA concentration and the monomer composition of the P(3HB-*co*-3HA) copolymer synthesized by the recombinant strain, 50 g/L of HSWP appeared to be the optical concentration for PHA production (1.0 g/L of PHA). In addition, the 3HB fraction in this copolymer was relatively high (80 mol%), which suggests that it would be expected to have good mechanical properties. The PHA production yield under the culture condition used here was estimated to be 20 mg-PHA/g-(H)SWP, which equates to approximately 1.0 g-PHA per liter of soybean wastewater. On the other hand, when HSWP, prepared using 5 N H_2_SO_4_, was used as both the nitrogen and carbon sources, the recombinant strain accumulated 26 wt% P(3HB-*co*-3HA) from 10–20 g/L of HSWP and the PHA concentration was 0.2–0.3 g/L (Table 4). Furthermore, the addition of more than 40 g/L of HSWP to the medium inhibited cell growth. This is probably due to the high concentration of salts formed by neutralization following hydrolysis. Thus, we found that both HSWP prepared using either 0.6 N or 5 N H_2_SO_4_ could be utilized as a nitrogen and carbon source, and HSWP prepared by hydrolysis with 0.6 N H_2_SO_4_ was suitable for cell growth and PHA production.

We also investigated PHA production using hydrolyzed cornstarch as a carbon source. As a result, 2.9 g/L of P(3HB-*co*-3HA) containing 86 mol% 3HB unit could be produced by the recombinant strain from 100 g/L of hydrolyzed cornstarch (Table 5).

Based on these data, we concluded that SWP and HSWP did not contain sufficient carbon to produce PHA, since the PHA contents (<35 wt%) obtained using HSWP were lower than the PHA content under control conditions (glucose and NH_4_Cl) (Table 2 and Table 3). Therefore, we attempted to add a mixture of SWP/HSWP and hydrolyzed cornstarch to the medium for PHA production (Table 6 and Table 7). After 72 h of cultivation, the recombinant strain accumulated 64 wt% P(3HB-*co*-3HA) containing 82 mol% 3HB unit from 10 g/L of SWP and 13 g/L of hydrolyzed cornstarch as nitrogen and carbon sources, respectively (Table 6). However, the dry cell weights (less than 0.7 g/L) and PHA concentration (less than 0.2 g/L) decreased when 26 g/L of hydrolyzed cornstarch was used. In the case when both HSWP, prepared using 0.6 N H_2_SO_4_, hydrolyzed cornstarch were added to the medium, cell growth was inhibited with increasing concentrations of hydrolyzed cornstarch (Table 7). The inhibition of cell growth is likely caused by the high concentration of salts formed by neutralization after hydrolysis. Therefore, the HSWP and hydrolyzed cornstarch should be desalted before use in PHA production. One alternative solution to this salt problem maybe through the use of enzymatic hydrolysis of SWP and cornstrach instead of acid hydrolysis treatment. Interestingly, the 3-hydroxyvalerate (3HV) unit was detected in the copolymer by GC analysis when HSWP was added to the medium (Table 3, Table 4 and Table 7). Hydrolysis of SWP may produce a substrate (e.g., fatty acids with odd numbers of carbon atoms) leading to the supply of the 3HV monomer. The reason for this remains unclear.

In conclusion, P(3HB-*co*-3HA) with various monomer compositions could be synthesized from SWP, HSWP, and/or hydrolyzed cornstarch as nitrogen and/or carbon sources in this study. However, the production efficiency was found to be unsatisfactory. Although SWP could be used as a nitrogen source, the PHA concentration was less than 1.0 g/L (less than 35 wt% PHA) when only HSWP was added to the medium as both a carbon and nitrogen source (Table 7). This suggests that SWP/HSWP contains sufficient nitrogen for the recombinant *Pseudomonas* sp. 61-3 strain to produce PHA, but it is deficient as a carbon source. Further improvements will therefore be necessary to achieve effective PHA production from SWP/HSWP. Since treatment of steamed soybean wastewater by a treatment facility, for example an activated sludge, is expensive, effective utilization of this wastewater is required. The production of PHA reported here is one proposed use of this wastewater. In the future, the molecular weight and mechanical properties of the copolymer synthesized should be further investigated since the molecular weight of the polymer affects its mechanical properties in addition to the monomer composition. The utilization of waste substrates, such as steamed soybean wastewater as a nitrogen and the carbon source, could contribute significantly to reducing the costs of PHA production as well as reducing the cost of waste treatment while at the same time promoting environmental conservation.

## Figures and Tables

**Table 1:** Nutrient composition of SWP (wt%).

Moisture	Crude Protein	Crude Fat	Fiber	Ash	Starch	Non-Fibrous Carbohydrate
12.37	10.91	0.17	0.13	12.83	1.75	61.84

**Table 2:** Biosynthesis of PHA *Pseudomonas* sp. 61-3 (*phbC*::*tet*) harboring pJKSc54-*phab* from SWP as the sole nitrogen source.

Nitrogen Source	Cultivation Time (h)	Dry Cell Weight (g/L)	PHA Content (wt%)	PHA Conc. (g/L)	PHA Composition (mol%)					
					3HB (C4)	3HHx (C6)	3HO (C8)	3HD (C10)	3HDD (C12)	3H5DD (C12’)
0.5 g/L NH_4_Cl	48	3.2	62	2.0	87	trace	2	6	2	3
1 g/L SWP	48	0.4	60	0.2	87	trace	1	7	2	3
	72	0.6	66	0.4	87	1	1	6	2	3
5 g/L SWP	48	0.9	52	0.5	80	trace	2	9	4	5
	72	1.2	55	0.7	81	1	2	8	4	4
10 g/L SWP	48	1.4	42	0.6	80	trace	2	10	4	4
	72	1.8	58	1.0	76	1	3	11	4	5
20 g/L SWP	48	2.4	39	1.0	76	1	2	11	5	5
	72	3.0	46	1.4	73	1	3	12	5	6
50 g/L SWP	48	4.4	23	1.0	78	trace	2	10	6	4
	72	5.5	32	1.8	68	1	4	14	7	6

Cells were cultivated at 28 °C for 48 or 72 h in nitrogen-free MS medium containing glucose (2%) and SWP (0.1, 0.5, 1, 2 or 5%) as the sole carbon and nitrogen source, respectively. SWP, soybean waste powder; 3HB, 3-hydroxybutyrate; 3HHx, 3-hydroxyhexanoate; 3HO, 3-hydroxyoctanoate; 3HD, 3-hydroxydecanoate; 3HDD, 3hydroxydodecanoate; 3H5DD, 3-hydroxy-*cis*-5-dodecenoate.

**Table 3:** Biosynthesis of PHA in *Pseudomonas* sp. 61-3 (*phbC*::*tet*) harboring pJKSc54-*phab* from HSWP (by 0.6 N H_2_SO_4_).

HSWP (g/L)	Dry Cell Weight (g/L)	PHA Content (wt%)	PHA Conc. (g/L)	PHA Composition (mol%)						
				3HB (C4)	3HV (C5)	3HHx (C6)	3HO (C8)	3HD (C10)	3HDD (C12)	3H5DD (C12’)
10	0.6	26	0.2	88	1	trace	1	5	3	2
20	1.3	33	0.4	85	1	trace	1	6	4	3
30	2.0	35	0.7	84	1	trace	2	7	3	3
40	2.6	33	0.9	81	1	trace	2	7	5	4
50	3.1	32	1.0	80	1	trace	2	8	5	4
75	4.0	32	1.3	76	1	trace	2	10	6	5
100	4.2	21	0.9	78	1	1	3	8	6	3

Cells were cultivated at 28 °C for 48 h in carbon- and nitrogen-free MS medium containing HSWP (10, 20, 30, 40, 50, 75 or 100 g/L) as the nitrogen and the carbon sources. SWP was hydrolyzed by 0.6 N H_2_SO_4_ at 80 °C for 5 h and neutralized by 5 N NaOH. HSWP, Hydrolyzed soybean waste powder; 3HB, 3-hydroxybutyrate; 3HV, 3-hydroxyvalerate; 3HHx, 3-hydroxyhexanoate; 3HO, 3-hydroxyoctanoate; 3HD, 3-hydroxydecanoate; 3HDD, 3-hydroxydodecanoate; 3H5DD, 3-hydroxy-*cis*-5-dodecenoate.

**Table 4:** Biosynthesis of PHA in *Pseudomonas* sp. 61-3 (*phbC*::*tet*) harboring pJKSc54-*phab* from HSWP (by 5 N H_2_SO_4_).

HSWP (g/L)	Dry Cell Weight (g/L)	PHA Content (wt%)	PHA Conc. (g/L)	PHA Composition (mol%)						
				3HB (C4)	3HV (C5)	3HHx (C6)	3HO (C8)	3HD (C10)	3HDD (C12)	3H5DD (C12’)
10	0.7	26	0.2	76	3	1	2	9	5	4
20	1.1	26	0.3	66	2	1	4	14	7	6
30	1.1	11	0.1	61	4	1	5	15	9	5
40	1.2	2	0.02	39	11	trace	5	21	19	5
50	0.9	2	0.02	42	16	trace	5	21	16	0
75	0	-	-	-	-	-	-	-	-	-
100	0	-	-	-	-	-	-	-	-	-

Cells were cultivated at 28 °C for 48 h in carbon- and nitrogen-free MS medium containing HSWP (10, 20, 30, 40, 50, 75 or 100 g/L) as the nitrogen and the carbon sources. SWP was hydrolyzed by 5 N H_2_SO_4_ at 90 °C for 1 h and neutralized by 5 N NaOH. HSWP, Hydrolyzed soybean waste powder; 3HB, 3-hydroxybutyrate; 3HV, 3-hydroxyvalerate; 3HHx, 3-hydroxyhexanoate; 3HO, 3-hydroxyoctanoate; 3HD, 3-hydroxydecanoate; 3HDD, 3-hydroxydodecanoate; 3H5DD, 3-hydroxy-*cis*-5-dodecenoate.

**Table 5:** Biosynthesis of PHA in *Pseudomonas* sp. 61-3 (*phbC*::*tet*) harboring pJKSc54-*phab* from hydrolyzed cornstarch.

Hydrolyzed Cornstarch (g/L)	Cultivation Time (h)	Dry Cell Weight (g/L)	PHA Content (wt%)	PHA Conc. (g/L)	PHA Composition (mol%)					
					3HB (C4)	3HHx (C6)	3HO (C8)	3HD (C10)	3HDD (C12)	3H5DD (C12’)
10 ^(1)^	48	0.2	2	0.004	34	0	0	25	17	24
	72	0.3	2	0.006	33	0	trace	25	15	27
100 ^(2)^	48	2.9	57	1.7	87	trace	1	6	3	3
	72	4.1	71	2.9	86	trace	2	6	3	3
14 ^(3)^	48	2.5	57	1.4	84	trace	2	7	4	3
	72	2.4	63	1.5	84	trace	2	7	4	3
20 ^(3)^	48	2.5	54	1.4	83	trace	2	8	4	3
	72	2.5	55	1.4	83	trace	2	8	4	3

Cells were cultivated at 28 °C for 48 or 72 h in MS medium containing 0.05% NH_4_Cl and hydrolyzed cornstarch (10, 14, 20 or 100 g/L) as the sole nitrogen and carbon source, respectively. ^(**1**)^ Cornstarch was hydrolyzed by 0.6 N H_2_SO_4_ at 80 °C for 5 h. ^(**2**)^ Cornstarch was hydrolyzed by 0.6 N H_2_SO_4_ at 80 °C for 8 h. ^(**3**)^ Cornstarch was hydrolyzed by 5 N H_2_SO_4_ at 90 °C for 1 h. 3HB, 3-hydroxybutyrate; 3HV, 3-hydroxyvalerate; 3HHx, 3-hydroxyhexanoate; 3HO, 3-hydroxyoctanoate; 3HD, 3-hydroxydecanoate; 3HDD, 3-hydroxydodecanoate; 3H5DD, 3-hydroxy-*cis*-5-dodecenoate.

**Table 6:** Biosynthesis of PHA in *Pseudomonas* sp. 61-3 (*phbC*::*tet*) harboring pJKSc54-*phab* from SWP and hydrolyzed cornstarch.

Hydrolyed Cornstarch (g/L)	SWP (g/L)	Cultivation Time (h)	Dry Cell Weight (g/L)	PHA Content (wt%)	PHA Conc. (g/L)	PHA Composition (mol%)					
						3HB (C4)	3HHx (C6)	3HO (C8)	3HD (C10)	3HDD (C12)	3H5DD (C12’)
13	1	48	0.4	55	0.2	78	1	3	10	4	4
		72	0.5	64	0.3	82	1	2	8	3	4
	10	48	1.1	35	0.4	74	1	2	12	6	5
		72	1.2	44	0.5	65	1	4	16	7	7
26	1	48	0.3	46	0.1	79	1	3	9	5	3
		72	0.3	43	0.1	72	1	3	12	8	4
	10	48	0.6	15	0.1	81	trace	2	9	5	3
		72	0.7	27	0.2	73	1	3	11	7	5

Cells were cultivated at 28 °C for 48 or 72 h in nitrogen-free MS medium containing SWP and hydrolyzed cornstarch as the nitrogen and the carbon source, respectively. Cornstarch was hydrolyzed by 5 N H_2_SO_4_ at 90 °C for 1 h. 3HB, 3-hydroxybutyrate; 3HV, 3-hydroxyvalerate; 3HHx, 3-hydroxyhexanoate; 3HO, 3-hydroxyoctanoate; 3HD, 3-hydroxydecanoate; 3HDD, 3-hydroxydodecanoate; 3H5DD, 3-hydroxy-*cis*-5-dodecenoate.

**Table 7:** Biosynthesis of PHA in *Pseudomonas* sp. 61-3 (*phbC*::*tet*) harboring pJKSc54-*phab* from HSWP and hydrolyzed cornstarch.

HSWP (g/L)	Hydrolyzed Cornstarch (g/L)	Cultivation Time (h)	Dry Cell Weight (g/L)	PHA Content (wt%)	PHA Conc. (g/L)	PHA Composition (mol%)						
						3HB (C4)	3HV (C5)	3HHx (C6)	3HO (C8)	3HD (C10)	3HDD (C12)	3H5DD (C12’)
5	20	48	0.5	47	0.2	80	1	trace	2	9	4	4
		72	0.6	56	0.3	75	1	1	3	11	5	4
	40	48	0.2	20	0.04	73	5	1	4	9	6	2
		72	0.2	22	0.04	73	3	1	4	9	7	3
10	10	48	1.0	51	0.5	79	1	1	2	9	4	4
	20	48	0.7	42	0.3	78	2	trace	2	9	5	4
50	0	48	2.2	34	0.7	85	1	trace	1	6	4	3
		72	2.3	39	0.9	84	1	trace	2	7	3	3
	15	48	0.04	0	0	0	0	0	0	trace	trace	0
		72	1.2	15	0.2	86	trace	trace	1	6	5	2
	30	48	0.02	1	trace	0	0	trace	0	100	0	0
		72	0.02	0	0	0	0	0	0	0	0	0

Cells were cultivated at 28 °C for 48 or 72 h in nitrogen-free MS medium containing the HSWP (5, 10 or 50 g/L) and cornstarch (0, 15, 20, 30 or 40 g/L) as the carbon and the nitrogen sources. Cornstarch was hydrolyzed by 5 N H_2_SO_4_. SWP was hydrolyzed by 0.6 N H_2_SO_4_. HSWP, Hydrolyzed soybean waste powder; 3HB, 3-hydroxybutyrate; 3HV, 3-hydroxyvalerate; 3HHx, 3-hydroxyhexanoate; 3HO, 3-hydroxyoctanoate; 3HD, 3-hydroxydecanoate; 3HDD, 3-hydroxydodecanoate; 3H5DD, 3-hydroxy-*cis*-5-dodecenoate.
